# Assessing land use change and the impacts on semi-natural habitats across England and Wales using four time points between 1930 and 2020

**DOI:** 10.1007/s10980-025-02189-8

**Published:** 2025-11-20

**Authors:** Lucy E. Ridding, Alexander R. Wickenden, Zephyr Orsler, Clare S. Rowland, Jennifer M. Hampton, Bruce Mitchell, Alistair Edwardes, Karen Mullin, Gavin Haughton, Neil Thurston, Ivano Pola, Geoffrey Sinclair, Mary-Rose Sinclair, Janet Shaw, Richard F. Pywell

**Affiliations:** 1 UK Centre for Ecology and Hydrology, Oxfordshire, Wallingford, OX10 8BB UK; 2 UK Centre for Ecology and Hydrology, Bailrigg, Lancaster, LA1 4AP UK; 3 Office for National Statistics, Newport, South Wales NP10 8XG UK; 4 Office for National Statistics, Titchfield, Hampshire, PO15 5RR UK; 5 Department for Environment Food and Rural Affairs, London, SW1P 4DF UK; 6 Wood PLC, Reading, RG2 9FW UK; 7 Environment Information Services, Pembrokeshire, Narberth, SA67 8AS UK; 8 Land Use Research Unit, King’s College, London, WC2R 2LS UK

**Keywords:** Agricultural intensification, Urbanisation, Grassland, Habitat loss, Restoration, Historical maps, Land use/land cover (LULC)

## Abstract

**Context:**

Habitat loss and degradation caused by human land use change is one of the major drivers of global biodiversity decline. Understanding historical patterns of land use/land cover (LULC) change over multiple time periods is essential for improving our knowledge of the magnitude and scale of habitat loss, but also for predicting future changes and targeting biodiversity conservation and restoration policy and actions.

**Objectives:**

This study assesses habitat loss resulting from LULC change in England and Wales between 1930 and 2020 at four different time points.

**Methods:**

We digitise a selection of published 1960s land use maps based on detailed field surveys, to use alongside existing published historical data (1930s) and more recent land cover datasets derived from satellite imagery (1990, 2020) for England and Wales.

**Results:**

Broadleaved woodland was the only semi-natural habitat to increase between the 1960s and 2020. Rough grassland, heath and wetland experienced the greatest loss between the 1930s and 1960s, predominantly through conversion to grassland. Grassland, which included species rich neutral grassland and agriculturally improved grassland was largely lost to arable land and this was greatest between the 1960s and 1990. This provides further evidence of post-war agricultural intensification as a key driver of habitat loss in England and Wales. Although this rate declined after 1990, it did not halt completely suggesting LULC change is still an important driver of biodiversity loss.

**Conclusions:**

The patterns revealed in this study may be used to predict where future land use changes are likely to occur or conversely where restoration of semi-natural habitats should be targeted. Knowledge of habitat loss over multiple time periods can increase the likelihood of restoration success since the location and timing of habitat destruction are both known.

**Supplementary Information:**

The online version contains supplementary material available at 10.1007/s10980-025-02189-8.

## Introduction

Habitat loss and degradation caused by human induced land use change is one of the major drivers of global biodiversity decline (Newbold et al. 2015; Jaureguiberry et al. 2022), which is predicted to continue over this century (Powers and Jetz 2019; Williams et al. 2021). The loss of habitats results in substantial range contractions and species extinctions, which continue to impact long after the destruction event (Chen and Peng 2017; Haddou et al. 2022). Many studies evaluating biodiversity loss examine change over more recent decades (Tracewski et al. 2016; Hallmann et al. 2017; Powney et al. 2019; Bowler et al. 2021) or use space-for time substitution (Yan et al. 2021; Attinello et al. 2024) and so the full extent of habitat loss and the rates of decline are not well understood. This information is essential for improving our knowledge on the magnitude and scale of loss, but also for predicting future changes and targeting conservation and restoration policies and actions to address land use change impacts on biodiversity.

Studies evaluating land use/land cover (LULC) change over time are often restricted by the availability of historic LULC data. Because of this many studies are only able to evaluate change for a specific study area or region (Cousins 2001; Bender et al. 2005; Hooftman and Bullock 2012) or for two recent snapshots in time (Trubins 2013; Meyer and Früh-Müller 2020). Whilst there are examples of LULC change evaluated over longer time scales (e.g. Levin et al. 2025) and nationally (e.g. Suggitt et al. 2023), the rates of change during the intermediate periods are often unknown. There are exceptions to this, for example, Winkler et al. (2021) examined global land use changes annually between 1960 and 2019, using a data-driven reconstruction approach, however such methods are often associated with large uncertainties (Bayer et al. 2017).

The UK has experienced dramatic intensification of land use over the twentieth century (Sinclair 1992; Swetnam 2007; Hooftman and Bullock 2012; Suggitt et al. 2023), which has led to a significant loss of semi-natural habitats (Hunting Surveys and Consultants Limited 1986; Fuller 1987; Webb and Vermaat 1990; Blackstock et al. 1995, 1999), as evident elsewhere in Europe (Farrell 1989; Cousins et al. 2015). These semi-natural habitats are the result of traditional extensive management such as grazing, coppicing, cutting or burning (Lake et al. 2014). In the UK, they include broadleaved woodland, heathland, semi-natural grassland/rough grazing and wetlands. They typically have a high conservation value, supporting a rich diversity of species and are also important for the delivery of several ecosystem services (Holland et al. 2017; Bengtsson et al. 2019; Stavi et al. 2022). Despite this, previous studies have indicated large losses in semi-natural habitats over the last 90 years (Moore 1962; Hodgson et al. 2005; Carey et al. 2008). For example, around 7% of ancient woodland present in England and Wales in the 1930s has been lost to other land uses, whilst 38% has been replaced with plantations, usually of non-native species (Spencer & Kirby 1992). During a similar period, semi-natural grasslands experienced a loss of 97% (Fuller 1987).

The decline in these semi-natural habitats across the UK has largely been attributed to agricultural intensification which occurred after the Second World War. This was largely driven by the 1947 Agriculture Act, which aimed to attain self-sufficiency in food production (Best and Coppock 1962) and continued to accelerate following the UK’s accession to the European Union (EU) in 1973. Large areas of semi-natural grassland underwent agricultural improvement, whereby swards were fertilised, limed and seeded with productive grass species, leading to species-poor swards with high biomass (Fuller 1987; Hopkins and Wainwright 1989; Poschlod and Wallis de Vries 2002). Grassland areas which were inaccessible or isolated were typically abandoned and reverted to scrub and woodland through natural succession (Poschlod and Wallis de Vries 2002). Wetlands were also targeted for conversion, though this occurred later, following improvements in drainage technology (Green 1990). Further semi-natural habitat losses were attributed to afforestation, which was a response to the depletion in the supply of timber during the First and Second World Wars. Initially most of the planting comprised of fast-growing conifers, however in the 1980s this shifted to using broadleaved trees (Mason 2007). Urbanisation increased rapidly during the twentieth century in the UK, as the population rose from ca. 46 million in 1930 to ca. 67 million in 2020 (Office for National Statistics 2024). Initially this occurred in towns, but the latter half of the twentieth century saw people moving away from the cities into rural areas (Clout 1986). Although the overall drivers of semi-natural habitat loss are known, the rate and pattern of the resulting LULC change is less well understood, together with their impacts on semi-natural vegetation.

The UK is fortunate to have a rich history in LULC data, providing an ideal opportunity to examine semi-natural habitat loss over multiple time periods. The first comprehensive land use survey of Great Britain took place in the 1930s (Land Utilisation Survey of Great Britain (LUSGB)) directed by Professor Dudley Stamp. This followed the great depression of British agriculture leading to the abandonment of arable land (Perren 1995). In the 1960s, a second survey in England and Wales was carried out by Professor Alice Coleman (Second Land Utilisation Survey (2LUS)), which built upon the original survey methodology. Although all the field surveys were completed for the 2LUS, only around 15% of the maps were published. Previously only small parts of these maps have ever been digitised (e.g. Swetnam 2007), however an extensive exercise was undertaken to analyse trends between LUSGB and 2LUS (Sinclair 1970, 1992; Coleman et al. 1974). This used systematic point sampling, based on the intersection points of a rectangular grid, which took over two-man years for a pilot area (2659 sq. km) (Coleman et al. 1974). More recently in the UK, land use datasets have utilised satellite imagery, with field survey data to train and validate classification methods. The first Land Cover Map (LCM) of Great Britain was produced in 1990 by the UK Centre for Ecology & Hydrology (Fuller et al. 1993), and a further eleven maps have been published bringing the series up to date at the time of writing (Morton et al. 2024).

In the UK, many LULC studies examine change over more recent decades, often regionally or using two snapshots in time. However, assessing patterns over the long-term and during intermediate time periods provides a greater understanding of how the rates and drivers of change vary through time. In this study we digitised the published 1960s land use maps based on field surveys, to use alongside existing historical data (LUSGB) for the 1930s and more recent land cover datasets (LCM1990, LCM2020), to understand change over 90 years at four different time points. We aim to address the following questions: (i) Does the rate of semi-natural habitat loss vary over time? (ii) How stable is the extent of semi-natural habitats over time? and (iii) Can we attribute the main drivers of semi-natural habitat decline over time?

## Methods

To investigate changes in LULC and semi-natural habitats across England and Wales, land cover maps were produced for 1930s, 1960s, 1990 and 2020. The extent of the study area was governed by the availability of published paper maps representing land use in the 1960s, which covered nearly 15% of the area of England and Wales (Figure S1). The extent and location of the published 1960’s maps had been based on where funding was available to support publication. Despite this, there is a reasonable representation of areas dominated by semi-natural habitats (e.g. South Wales), urban (e.g. Southern England) and agricultural land (e.g. Midlands, North-East England, East Anglia). To test this, a comparison of LULC coverage within the range of the published maps was contrasted with the total area of England and Wales using 2020 as a proxy (Table S2). The majority of LULC types represented a similar area in both the 2LUS extent and the total area of England and Wales (± 2%). The only exceptions were grassland, which was slightly underrepresented in the 2LUS maps (-5.0%) and urban which was overrepresented (+ 8.9%), most likely due to several of the maps being published around Greater London. The following sections detail the datasets used for each time period.

### 1930s

The Land Utilisation Survey of Great Britain (LUSGB) was the first comprehensive survey of land use in Great Britain. It was directed by Professor L. Dudley Stamp of the London School of Economics in the 1930s and 1940s. Mapping was carried out by volunteers, including university students and school children who labelled six-inch maps with six different land use categories in the field. The land use groups included: (a) meadow and permanent grass, (b) arable land including rotational grass, (c) heathland and moorland or rough pasture, (d) forests and woodland, (e) gardens, including orchards and allotments (f) agriculturally unproductive land and (g) inland water (see Stamp 1931 for further details). The 175 maps were published between 1933 and 1949, at one inch to the mile (1:63,360) scale and in 2003/4 these were digitally scanned and geo-referenced. These were later vectorised using a semi-automated supervised classification (see Baily et al. 2011; Entec 2010).

### 1960s

In the 1960s, a second survey of Great Britain, the “Second Land Utilisation Survey” (2LUS) was directed by Professor Alice Coleman of King’s College London (Coleman 1961). A similar approach using volunteers was employed (though some were paid), however in this survey more detailed land use information was collected with the aim of publishing at the finer scale of 1:25,000. Although the fieldwork was completed by around 3000 volunteers, only 118 of the survey maps were published in England and Wales (plus one in Scotland), each covering 200 km^2^. Note this also included one replicate for Princes Risborough. Seventy land use categories were mapped at two different levels of intensity (Coleman 1964). The first level consisted of thirteen groups, each represented by a distinctive colour, summarised in Table 1. The second level were subdivisions of the main thirteen groups represented by tone variations of the main colour. For example, arable land was split into six subdivisions; ley legumes, cereals, root crops, green fodder, industrial crops and fallow (Coleman 1968; Coleman and Maggs 2024).

**Table 1:** Classified land use categories mapped in the 1960s Second Land Utilisation Survey

2LUS Land use category	Colour	Digitisation status
Arable	Light brown	Classified
Market gardening	Purple	Classified together with Orchards
Orchards	Purple stripes	Classified together with Market gardening
Woodland	Dark green	Classified and manually split into broadleaved and coniferous
Heath, moorland and rough land	Yellow	Classified
Grass	Light green	Classified
Open spaces	Lime green	Classified
Water and Marsh	Light blue	Classified
Unvegetated land	White	Manually digitised, with coastal areas separated into a “coastal” category
Industry	Red	Classified (later merged with Settlement)
Transport	Orange	Classified (later merged with Settlement)
Settlement	Grey	Classified
Derelict Land	Black stipple	Not classified but grouped with Settlement

As part of this current study, we digitised 110 of the published 2LUS maps in England and Wales that we had access to (eight were missing from the archive) using a semi-automated supervised classification method, consistent to the approach used for the LUSGB. Firstly, maps were scanned and saved as Tiff images at 300 dots per inch at a colour depth of 8 bits per channel (24 bit depth). These were georeferenced in ArcGIS v10.7 using the Ordnance Survey (OS) 1 km National Grid (see Supplementary Material S3 for further details). For the classification, georeferenced 3-band images were converted to 16-bit single band images (256 colours). A manual look-up table was created for each map image identifying the dominant unique pixel values of the different land use classes. Any “noise” present on the images including text, contour lines and symbols were assigned a single blank value of 0. Similarly, pixel values which were common to multiple land use classes, such as the white colour were also assigned to 0. For these reasons it was not possible to classify either the “unvegetated land” and “derelict land” land use categories. The model reclassified the 256 colours within a range of 0 to 10, representing the 10 land use categories and the noise category (pixel value = 0), with the latter being removed and replaced. Classified images were vectorised and manually cleaned using the eliminate tool in ArcGIS. All “unvegetated land” was manually digitised as this could not be classified, along with a new category for “coastal” areas which were not classified. Railway lines, roads and very small rivers were inconsistently classified and were therefore removed, while “industry” and “transport” classes were grouped with “settlement” to ensure uniformity. “Derelict land” could not be classified but was often mapped in the classification as “settlement”, so this was later grouped with this category. Woodland was manually split into broadleaved and coniferous using the woodland symbology provided on the OS background, this usually aligned with the manual over-printed symbols but the latter was not present for all woodlands, thus the OS backdrop was used. The date of each OS basemap varied, however these were largely published or reprinted in the 1960s and were largely consistent with the surveyed land cover data, though there were exceptions e.g. Bristol East.

To validate the classified 2LUS maps, 3,000 random points were generated across the total area covered by the 110 maps. For each point, a manual assessment was undertaken assigning the most representative land cover class to the point based on the raw georeferenced 3-band images. A confusion matrix between the manually assigned points and the classified 2LUS maps revealed an overall accuracy of 95.8% (Kappa = 0.94) (see Supplementary Material 3 for detailed methodology and results).

### 1990 and 2020

For 1990 and 2020, we used the UK Centre for Ecology & Hydrology’s Land Cover Maps (LCM) for 1990 (Rowland et al. 2020) and 2020 (Morton et al. 2021) at 25 m spatial resolution. These products are based on the classification of satellite and contextual spatial data (e.g. a digital elevation model for slope and aspect, coastline data etc.) (Marston et al. 2023), and map 21 land cover classes, based on a nomenclature corresponding to the UK Broad Habitats (Jackson, 2000). The LCM1990 replaces the earlier Land Cover Map of Great Britain (LCMGB) (Fuller et al. 1993) produced in the early 1990’s, to ensure the methodology used to create the LCM1990 and LCM2020 was consistent.

### Combining the LULC time-series

To enable an accurate comparison of LULC and habitats over time, we pooled the range of categories across each of the four datasets into the following aggregate categories: “broadleaved woodland”, “coniferous woodland”, “rough grassland, heath and wetland”, “grassland”, “arable and horticulture”, “freshwater”, “urban” and “other”. It was difficult to differentiate semi-natural grasslands from agriculturally improved grassland (intensively managed highly productive grasslands which have low biodiversity value) in the historical maps of 1930s and 1960s, since these were mapped together. In 1930, agriculturally improved grassland would have occurred at very low levels (Fuller 1987; Hooftman and Bullock 2012), therefore most of the grassland would align with what we classify as neutral grassland today. This was verified using an independent vegetation dataset recorded in the 1930s by Professor Ronald Good across Dorset, Southern England (Good 1937). In the 1960s, grassland was mapped as “grassland” and “open space”. The latter was described as “non-productive space, excluding agricultural land on the one hand, and untended heath and rough land on the other” (Coleman 1968), so included private parkland, recreational areas and golf courses etc. This therefore aligns more closely with modern day improved grassland. The “Grassland” category was typically challenging to map during the 1960s 2LUS field campaign, since it relied heavily upon the timing of the mapping undertaken by the surveyor (Coleman 1968). “Grassland” therefore included a mixture of grassland habitat quality, which we would categorise today as semi-natural and agriculturally improved grassland. To verify this, we overlaid an independent dataset of grassland vegetation surveys undertaken across England in the 1960s, that were used in the Nature Conservation Review (NCR) (Ratcliffe 1977), which aimed to identify the most important places for nature conservation in Great Britain (Ridding et al. 2015). Of the 205 neutral grassland verification points, 80% of these overlapped with the 1960 2LUS category of “grassland”. We also used this dataset to verify that other semi-natural grasslands such as calcareous grassland and acid grassland were correctly mapped as rough grasslands under “heath, moorland and rough land” in the 1960s 2LUS maps.

The historical maps of the 1930s and 1960s did not map the coastal categories to the same detail found in 1990 and 2020 land cover maps (littoral and sublittoral classes). Furthermore, some freshwater areas mapped in the 1930s and 1960s were manually reclassified to match the seawater classified in 1990 and 2020. For these reasons, the “other” broad category became broader than envisioned, and also included “unvegetated land” (1960s) and “inland rock” (1990 and 2020), categories not mapped in the 1930s. Some marsh/wetland habitat in very close vicinity to rivers would have been classified as water in 1960 rather than “rough grassland, heath and wetland”, as these were mapped with the same colours and were therefore classified together. Additionally, it was not possible to separate woodland into broadleaved and coniferous in the 1930s. Further cleaning of the 1930s maps was also undertaken, including the removal of roads, to ensure consistency between the 1960 map, and the later land cover datasets.

The historical vector maps for the 1930s and the 1960s were converted to a 25 m × 25 m raster in ArcPro v3.4 to match the pixel size of the LCM1990 and LCM2020. To ensure a consistent extent was evaluated between the time periods, we masked each of the rasters by the Mean High Water mark coastline boundary (Office for National Statistics 2023). If any remaining cells within the rasters contained no data, they were removed from the corresponding years to allow for a uniform comparison. This left an area of 18,891 km^2^ across England and Wales to examine for semi-natural habitat change over time.

After producing the final LULC maps at 25 m resolution for each of the four time periods, we calculated the total area of each land cover class in the 1930s, 1960s, 1990 and 2020 to understand how the overall extent of semi-natural habitats and LULC had changed over time across the study area. We also repeated this analysis at a regional level to understand spatial patterns across England and Wales. Five regions were assessed based on combining the Government Office Regions: Midlands (East and West), North of England (including Yorkshire and the Humber), South of England (including London), East of England and Wales (Office for National Statistics 2025). To understand the stability of semi-natural habitats (“rough grassland, heath and wetland”, “grassland” and “broadleaved woodland”) and LULC more widely over time, we determined the extent of area where land cover classes remained consistent between 1930 and 2020. To do this we extracted each land cover class individually starting with 1930 and produced a transition matrix for the class to determine which cells had stayed the same in 1960, then again in 1990 and 2020. We also investigated the fate of lost semi-natural habitats in the 1960s, 1990 and 2020 using transition matrixes for the semi-natural habitat classes to understand the main drivers of change over time. This was assessed as the proportion of lost “rough grassland, heath and wetland” and “grassland” broad categories during each time point, since the latter also contained semi-natural grassland, as well as improved grassland (Table 2).

**Table 2:** Scheme used to match land cover classes from each of the four datasets: 1930s (Land Utilisation Survey of Great Britain), 1960s (Second Land Utilisation Survey), 1990 (Land Cover Map 1990) and 2020 (Land Cover Map 2020)

Broad category	UKHab codes	1930s	1960s	1990/2020
Broadleaved woodland	w1	Woodland	Broadleaved woodland	Broadleaved woodland
Coniferous woodland	w2		Coniferous woodland	Coniferous woodland
Rough grassland, heath and wetland	g1, g2, h1, h2, h3, f1, f2	Rough Grazing, Heathland, Moorland	Heath, moorland and rough land	Calcareous grassland
				Acid grassland
				Fen, Marsh and Swamp
				Heather
				Heather grassland
				Bog
Grassland	g3, g4, c1b	Meadowland, Permanent Grass	Open space	Improved Grassland
			Grassland	Neutral grassland
Arable and horticulture	c1a,c,d,e	Arable	Arable	Arable and horticulture
		Orchard	Orchard	
			Market garden	
Urban	u1	Urban	Settlement	Urban
		Suburban	Industry	Suburban
			Transport	
			Derelict land	
Freshwater	r1, r2	Water	Water and marsh	Freshwater
Other	t1, t2, s1, s2, s3	Seawater (manual)	Unvegetated land	Inland rock
			Coastal (manual)	Saltwater
			Seawater/marsh (manual)	Supra-littoral rock
				Littoral rock
				Supra-littoral sediment
				Saltmarsh
				Littoral sediment

The pooled categories used in this study can be found in the first column, alongside the corresponding UK Habitat Classification code (UKHab Ltd 2023).

## Results

### Change in LULC and semi-natural habitats

We revealed substantial changes in LULC between 1930 and 2020 across England and Wales (Fig.s 1 and 2). The greatest decline was identified for grassland, with a net loss of 2214 km^2^. The largest loss of grassland occurred between the 1960s and 1990 (− 21%), with smaller losses also evident between the 1930s and 1960s (− 3%) and 1990 and 2020 (− 7%) (Table 3). Trends in regional differences were more variable (Figure S2), which likely reflects the mix of species-poor improved grassland and neutral grassland present across England and Wales. Considerable losses were also identified for semi-natural habitats represented by the rough grassland, heath and wetland broad category. The largest decline occurred between the 1930s and 1960s (− 26%), followed by the 1960s and 1990 (− 20%) and 1990 and 2020 (− 11%), resulting in a net loss of 1133 km^2^. The declining trend was evident across all five regions, with the exception of Wales in 1990, where rough grassland, heath and wetland increased slightly. Freshwater was the only other broad category to decline over the study period with a smaller loss of 152 km^2^, however some of this change is likely to reflect differences in the mapping of rivers between the historical maps (1930s and 1960s) and the satellite derived maps of 1990 and 2020. Due to its linearity, freshwater is typically more challenging to map at this scale. This is also an issue for other LULC types e.g. thin strips of remnant semi-natural grassland on steep slopes.

**Fig. 1:**
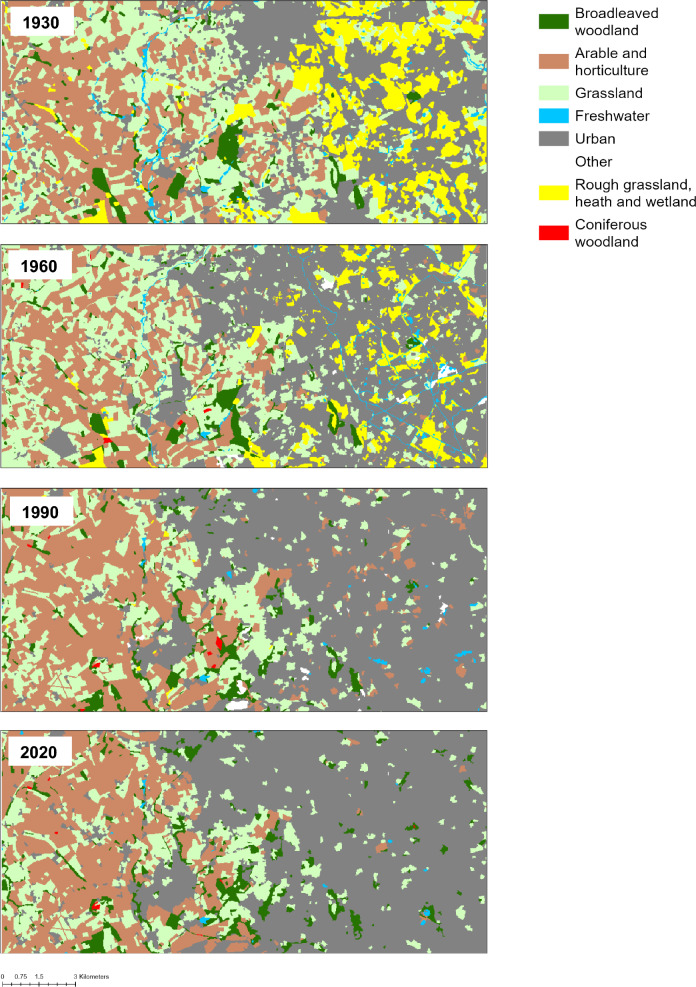
Land cover maps for an example area (Wolverhampton South, England) in the 1930s, 1960s, 1990 and 2020

**Fig. 2:**
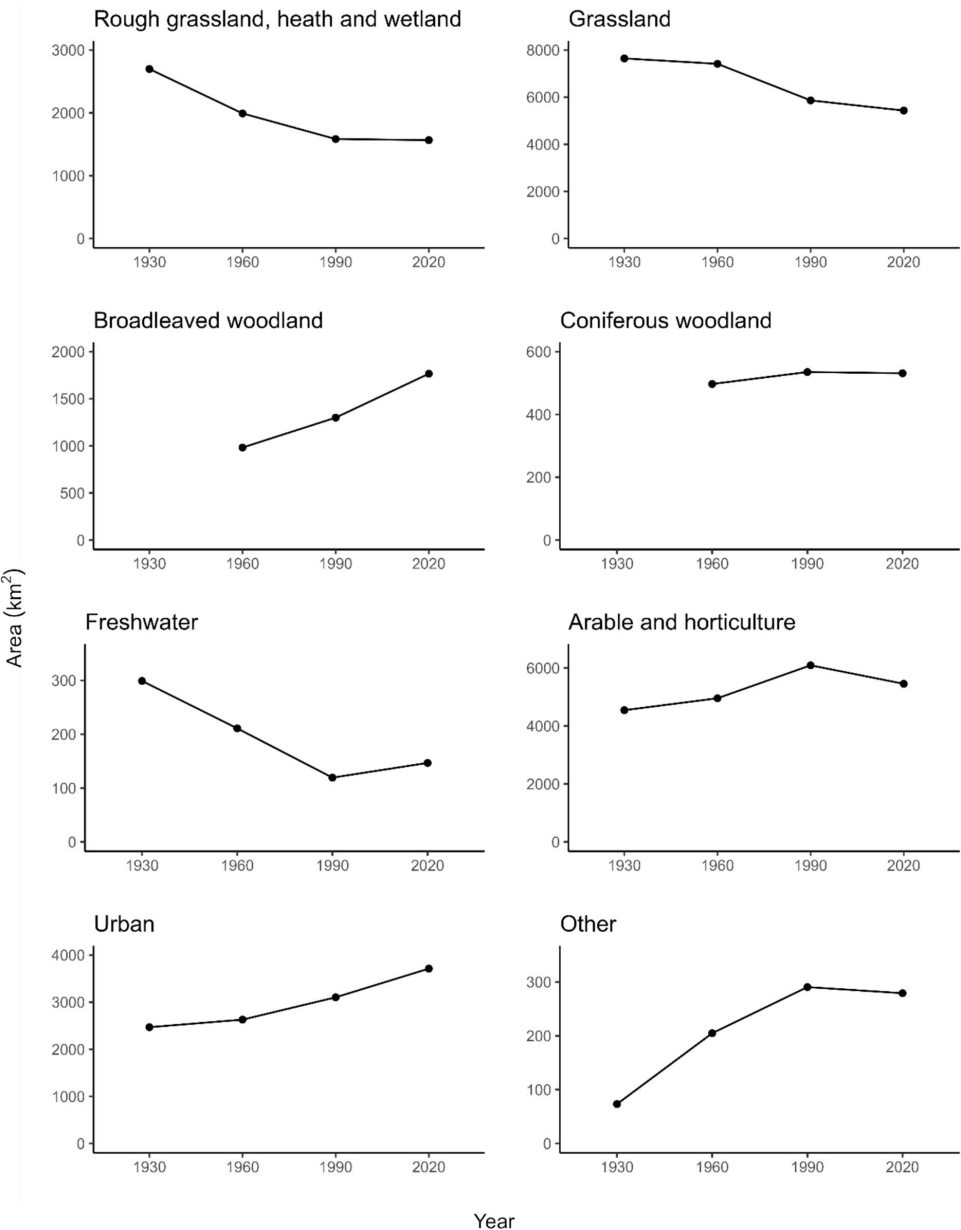
Area of the eight land cover classes across the study region in England and Wales in the 1930s, 1960s, 1990 and 2020. In 1930, broadleaved woodland and coniferous woodland are excluded, since these could not be distinguished

**Table 3:** Area (km^2^) and proportion (%) of each broad category within the study area across England and Wales in the 1930s, 1960s, 1990 and 2020

	1930s	1960s	1990	2020
Broadleaved woodland	1158.17 (6%)	982.20 (5%)	1299.92 (7%)	1766.06 (9%)
Coniferous woodland		497.14 (3%)	535.19 (3%)	531.37 (3%)
Rough grassland, heath and wetland	2699.35 (14%)	1991.70 (11%)	1583.80 (8%)	1566.45 (8%)
Grassland	7647.31 (41%)	7417.61 (39%)	5864.59 (31%)	5433.09 (29%)
Arable and horticulture	4544.79 (24%)	4956.69 (26%)	6093.89 (32%)	5454.98 (29%)
Urban	2469.14 (13%)	2629.86 (14%)	3103.67 (16%)	3713.10 (20%)
Freshwater	299.07 (2%)	210.95 (1%)	119.38 (< 1%)	146.71 (1%)
Other	73.19 (< 1%)	204.86 (1%)	290.59 (2%)	279.24 (2%)

NB in 1930 broadleaved woodland and coniferous woodland are combined.

Increases were evident in both broadleaved and coniferous woodland, as well as urban cover and arable and horticulture (Fig. 2). The greatest proportional change was evident for urban land which increased from 13% cover of the study area in 1930 to 20% in 2020, resulting in a net increase of 1243 km^2^. This increasing trend was largely consistent across all five regions (Figure S2). Large gains were also revealed for arable and horticulture, which increased the most during the 1960s and 1990 to reach a peak in 1990 (32% of the study area), after which there was a decline (− 10%). The peak in 1990 was particularly pronounced in the north of England compared with other regions (Figure S2). It was not possible to determine the extent of broadleaved woodland and coniferous woodland in the 1930s, but overall woodland increased from 6.1% of the study area to almost double in 2020 (12.1%). Individually, both woodland types increased between the 1960s and 2020, albeit to a much lesser extent for coniferous woodland (net gain of 34 km^2^), whilst broadleaved woodland increased by 910 km^2^. The patterns observed for broadleaved woodland were similar across regions, however between 1990 and 2020, Wales experienced an increase in coniferous woodland, whilst in the north of England coniferous woodland declined (Figure S2). Small areal increases were identified for the other broad category representing coastal land covers and sparsely vegetated areas, however this could reflect the lack of sparsely vegetated land cover which was not captured in the 1930s maps.

### Stability of LULC

**Fig. 3:**
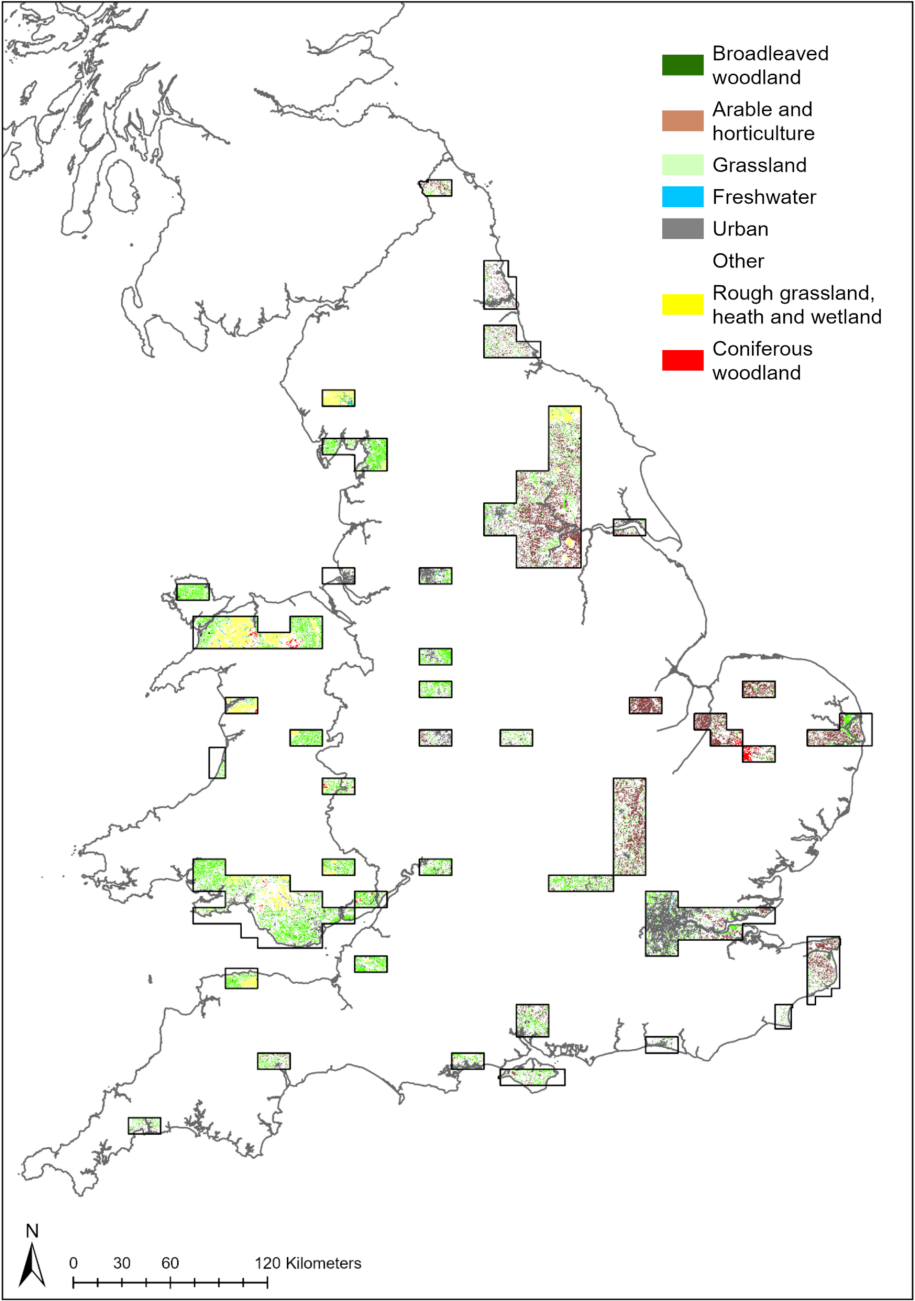
Spatial extent of the 8 broad categories which have remained consistent in the 1930s, 1960s, 1990 and 2020 across England and Wales. Since broadleaved woodland and coniferous woodland could not be distinguished in the 1930s, we assumed that if the woodland type consistently appeared in the 1960s, 1990 and 2020, this was also the woodland type in the 1930s

Only 40% of the study area had a stable LULC between the 1930s and 2020 at the scale analysed, whereby the land cover designated in 1930 remained consistent in the 1960s, 1990 and 2020 (Fig. 3). This means an area of 11,334.6 km^2^ has changed at least once between the 1930s and 2020 across the study area in England and Wales. The greatest stability was found for grassland and arable land where 13% and 11% of the study area remained stable, respectively. Grassland was largely stable in southern Wales, whilst there was greater stability for arable land in northern and eastern England. Only 5% of the area of semi-natural habitats (excluding neutral grassland) did not change to another LULC between any of the evaluated time periods. Stability for these habitats was greatest in northern Wales and parts of northern England (Fig. 3).

### Conversion of semi-natural habitats

**Fig. 4:**
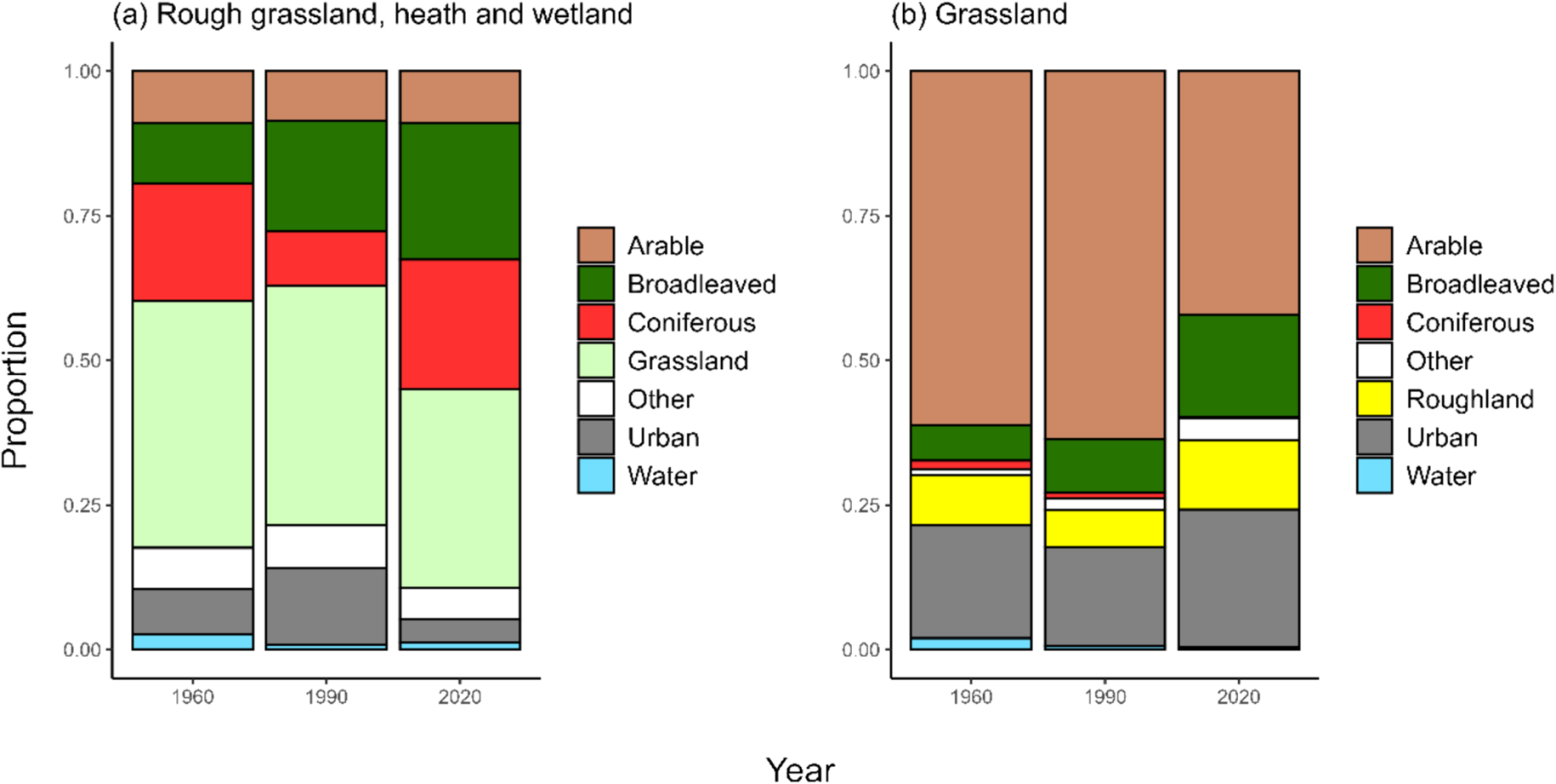
The proportion of (**a**) rough grassland, heath and wetland and (**b**) grassland converted to other broad categories (arable and horticulture, broadleaved woodland, coniferous woodland, grassland, other, urban and water) in the 1960s, 1990 and 2020 across England and Wales

The largest proportion of rough grassland, heath and wetland was lost to grassland in the 1960s (43%), 1990 (41%) and 2020 (34%) (Fig. 4). A considerable proportion was also lost to woodland. In the 1960s more rough grassland, heath and wetland was lost to coniferous woodland, whereas in 1990 a greater proportion was lost to broadleaved woodland. In 2020, a similar proportion was lost to both broadleaved and coniferous woodland. Conversion to arable land was consistently lower in each of the three time points (9%), whilst urbanisation was greatest in 1990 (13%). Conversely for the broad grassland category, which contained both semi-natural neutral grassland and agriculturally improved grassland, the greatest losses were to arable land across all three time periods. Urbanisation was also responsible for grassland loss, accounting for 20% in the 1960s, 17% in 1990 and 24% in 2020. Smaller losses to broadleaved woodland were also evident with the greatest proportional loss occurring in 2020 (18%). A small proportion of grassland was classified as rough grassland, heath and wetland in the 1960s, 1990 and 2020.

## Discussion

### Changes in semi-natural habitats

This study revealed considerable declines in semi-natural habitats across England and Wales between 1930 and 2020. Over the 90-year period we identified a net loss of 3347 km^2^ of rough grassland, heath, wetland and grassland (neutral and improved) from the studied landscape (18%). This value is likely to be a large underestimate of semi-natural grassland loss, since species-rich neutral grassland could not be separated from species-poor improved grassland in this study. Since most grassland in the 1930s was considered species-rich (Good 1937; Fuller 1987), it is highly likely that the loss of neutral grassland is masked by conversion to agriculturally improved grassland from the 1960s and onwards in this study. For instance, Fuller (1987) reported a loss of 97% between 1930 and 1984 in England and Wales, whilst Ridding et al. (2015) revealed a loss of 47% of the studied semi-natural grasslands in England between the 1960s and 2013. Both studies find significant losses of neutral grassland to agriculturally improved grassland, which could not be captured in this current study. Furthermore, our study area did not incorporate many lowland landscapes in southern England, which are known to have undergone dramatic losses of semi-natural habitats (Burnside et al. 2003; Hooftman and Bullock 2012). On the other hand, broadleaved woodland was the only semi-natural habitat to increase between the 1960s and 2020 across all five regions. This increase is consistent with trends across Great Britain, as monitored by the National Inventory of Woodland and Trees (Smith and Gilbert 2003). The greatest increase in broadleaved woodland occurred between 1990 and 2020 in this study, which coincides with the introduction of the 1985 Broadleaves Policy, which aimed to maintain and increase broadleaved woodland in the UK.

### Timing of LULC change

This study identified two distinct patterns of loss of semi-natural habitats, namely a dramatic loss of rough grassland, heath and wetland to productive grassland between the 1930s and the 1960s, and a later loss of grassland (including species-rich neutral grassland) to arable land that occurred between the 1960s and 1990. These losses slowed dramatically between 1990 and 2020. These patterns are consistent to those revealed in Dorset, a predominantly rural county in southern England evaluated over a similar time period (Ridding et al. 2020c). They found non-linear declines for most semi-natural habitats, with the greatest losses occurring between 1950 and 1980, after which the decline decreased. This may be due to the designation of Sites of Special Scientific Interest (SSSIs), the basic unit of statutory protection in England and Wales, which strengthened protection over time. The first sites were established in the 1950s, under The National Parks and Access to the Countryside Act 1949. These were later enhanced following the introduction of the Wildlife and Countryside Act 1981, and again after the Countryside and Rights of Way Act 2000, which increased power to refuse consent for damaging activities (DEFRA 2009). Other studies have demonstrated the importance of SSSIs for retaining semi-natural habitats and rare species (Ridding et al. 2015; Cooke et al. 2023). The decline in arable land across England and Wales between 1990 and 2020, consistent with Bibby (2009) is likely to reflect increases in farm mechanisation and farming intensity, including the use of pesticides and fertilisers. This would make it more difficult and less profitable to farm more marginal arable land, such as smaller fields on steep gradients with thin or wet soils, which would therefore have reverted to improved grassland (Robinson and Sutherland 2002; Zayed and Loft 2019). The declining trend in agricultural land is consistent with trends across Europe (Bičík et al. 2015; van Vliet et al. 2015), though this varies spatially (Kuemmerle et al. 2016).

### Drivers of semi-natural habitat decline

Most rough grassland, heath and wetland was lost to grassland within each of the time periods assessed, whereas most grassland was consistently lost to arable. This provides further evidence for agricultural intensification as a key driver of habitat loss, as found in other studies across England and Wales (Fuller 1987; Hooftman and Bullock 2012; Ridding et al. 2015). The results also support the notion that neutral grasslands were easier to convert to arable land compared with other semi-natural habitats, such as calcareous grassland, that often occurs on steep slopes, or wetlands that would require drainage prior to conversion (Green 1990; Hodgson et al. 2005). Instead, these areas were converted to improved grassland through reseeding and fertiliser application. Additionally, some rough grassland may have been lost to improved grassland indirectly through inappropriate grazing management and eutrophication caused by nitrogen deposition (Maskell et al. 2010; Newton et al. 2012; Ridding et al. 2020a), which has affected grasslands globally (Stevens et al. 2022). There was also some evidence of grassland converting to rough grassland, heath and wetland across the study period. Some of this may arise due to inaccuracies when differentiating between grassland types. For instance, in the 1960s, grasslands were challenging to distinguish on the ground depending on the season (Coleman 1968). Several grassland locations were resurveyed and checked in the field by co-author Geoffrey Sinclair, leading to corrections on some field sheets. Whilst for 1990 and 2020, differentiating grassland types using satellite imagery can be difficult (Marston et al. 2023). However, some of this change may arise due to areas of improved grassland undergoing restoration back to semi-natural grassland through the introduction of appropriate management (Crofts and Jefferson 1999). For instance, Wales was the only region to reveal an increase in rough grassland, heath and wetland between 1990 and 2020 in this study.

Both rough grassland, heath and wetland, and grassland categories were also lost to broadleaved woodland. This is likely to be the result of lack of management and abandonment, whereby grassland and heathland are converted to woodland through natural succession (Rose et al. 2000; Poschlod and Wallis de Vries 2002). The proportion of both broad categories lost to broadleaved woodland is greatest in the latter time period, which may reflect the lengthy process of invasion by shrubs and conversion to scrub before becoming a secondary woodland (Crofts and Jefferson 1999). Conversely, some areas may have been lost to broadleaved woodland through sustainable forest planting, as discussed previously. The proportion of rough grassland, heath and wetland lost to coniferous woodland was much higher compared with grassland. This is likely because afforestation was an important driver of change on heathlands during the twentieth century in the UK (Webb 1986). Despite this, the area of coniferous woodland was fairly consistent across the studied landscape between the 1960s and 2020, suggesting an increase in coniferous woodland in some areas, whilst others were lost to harvesting and replaced by broadleaved planting (note the Broadleaved woodland category also includes mixed woodlands). It is possible that in the 1960s there was some confusion between broadleaved woodland and coniferous woodland, that required manual digitisation (Supplementary Material 3). Furthermore, the key period of conifer planting, which occurred after the Second World War (Aldhous 1997; Raum 2020), could not be examined due to woodland types being grouped in the 1930s maps.

There was also evidence of urbanisation on both rough grassland, heath and wetland and grassland, albeit a slightly greater proportion in grassland. This loss was fairly consistent between the 1930s and 2020, across all five regions which reflects the increase in population over time and the demand for housing, leading to the various policy drivers which encouraged house building during this period (e.g. Wheatley Housing Act 1924, New Towns Act of 1946) (Metcalfe 2024). Similar trends in population size and urban land are also evident across Europe, where built-up surfaces expanded by almost 1,000 km^2^ per year between 2000 and 2015 (Schiavina et al. 2022).

It is important to remember that urban areas were overrepresented in the 2LUS extent used in this study, thus patterns in urbanisation presented in this study may be overestimated for England and Wales.

### Implications for biodiversity

This study has revealed dynamic changes in land use over 90 years, with around 60% of the study area changing at least once during this time. This is considerably higher than the estimated area globally (17%), albeit over a later time period (1960–2019) (Winkler et al. 2021). This has important implications for biodiversity loss not only through the direct loss of habitats, but also through habitat degradation, fragmentation, and changes to the surrounding matrix. When a habitat is lost, species are also lost, since smaller habitats support fewer species according to the species-area relationship (Arrhenius 1921). However, habitat fragmentation can lead to a reduction in connectivity, as well as increasing edge effects which can impact species abundance and distribution (Fahrig 2003; Zipkin et al. 2009; Willmer et al. 2022), though some of these effects are disputed (Fahrig et al. 2019). The area surrounding remaining habitat fragments, the matrix, also plays an important role in connecting habitats and enabling species movement (Ricketts 2001; Prevedello and Vieira 2010). Thus, even where the habitat itself has not been lost, if the surrounding matrix is not stable over time this can further exacerbate the impacts on biodiversity (Hooftman et al. 2016). For example, in one of our study areas (Fig. 1), we see areas of rough grassland, heath and wetland patches that remain between the 1930s and 1960s, however much of the surrounding area has been converted to urban land. Finally, land use change can also indirectly impact biodiversity, through influencing other environmental processes, including changes in atmospheric composition and subsequently climate change (Findell et al. 2017).

The peak timing of habitat loss in this study also has important implications for determining the impact on biodiversity loss, since many long-term biodiversity studies evaluate change over the last thirty to forty years. In this study, semi-natural habitats had already undergone significant loss by this point, thus, our understanding of past land use change as a driver of biodiversity decline is still limited. One exception is Suggitt et al. (2023) who found that the combined impact of climate change and land conversion increased extinction risk for 12% of species and reduced extinction risk for 40% between 1930 and 2007 in Great Britain. Although the loss of semi-natural habitat declined after 1990 in this study, the impact on biodiversity through delayed extinctions, termed the extinction debt (Tilman et al. 1994; Kuussaari et al. 2009) is likely to continue after habitat destruction events, as evident in Dorset over similar time scales (Ridding et al. 2020b) and across Europe (Schtickzelle et al. 2005; Saar et al. 2012).

It is important to acknowledge that LULC change studies such as this, are associated with uncertainties, due to temporal and spatial consistency issues arising from the use of different data sources and methodologies (Verburg et al. 2011). Whilst we have aimed to minimise these through scaling, aggregating classes and undertaking validation, some issues still remain and should be treated with caution. For example, change in freshwater and the other broad category are likely to be underestimated and overestimated respectively through time, due to inconsistencies in their mapping, particularly for smaller linear features such as rivers (for further details see Table S4). These maps should therefore not be used to assess local change, however when considered at the broader scale, these datasets provide a powerful insight into habitat loss over time. It is also important to remember that although the distribution of sites corresponded well to the overall LULC types found across England and Wales (Table S2), the sites were not selected by stratification and instead offer insight to LULC change over a range areas across England and Wales.

## Conclusion

This study reveals the extent of habitat loss and the highly dynamic nature of LULC in England and Wales for four time periods over 90 years. We can attribute these changes to the known drivers of habitat loss. The addition of the digitised 2LUS maps was critical for enhancing our understanding of these patterns, by providing insight into a key time period of LULC change, that was otherwise unquantified. We find the greatest loss of rough grassland, heath and wetland between the 1930s and the 1960s, predominantly to grassland, whilst more grassland was lost to arable between the 1960s and 1990. This provides further evidence of agricultural intensification as a key driver of habitat loss in the UK. Although this loss declined after 1990, it did not halt completely suggesting land use change is still an important driver of biodiversity loss.

Future research could directly assess the impact on biodiversity over time by incorporating modern and historical species records for well-studied taxa such as birds and butterflies. This would build on work by Suggitt et al. (2023), by allowing the relationship between species diversity and LULC change to be examined during multiple intermediate time periods. Future research could also explore the relative importance of habitat area and configuration on biodiversity loss over time by assessing landscape metrics such as patch size, shape, proximity/isolation (Wang et al. 2014), as well as the surrounding matrix as mentioned previously.

The findings of this study have important implications for land management and habitat restoration in the future. The patterns revealed may be used to predict where future land use changes may occur or conversely where restoration or re-creation of semi-natural habitats could be carried out to maximise the benefits for biodiversity. For example, by targeting restoration to locations where the habitat previously occurred in the recent past. The latter is important, since habitats lost more recently in time are likely to have a greater chance of restoration success before populations of key species become extinct (Dobson et al. 1979). Assessing multiple time periods, rather than examining two snapshots in time, as seen in this study, therefore enables a more accurate picture of when the habitat is likely to have been lost. The urgency to restore habitats has been recognised by the United Nations, who declared 2021–2030 as the Decade on Ecosystem Restoration (United Nations 2025). Internationally legally binding targets have been developed, for example the Nature Restoration Law aims to restore 20% of EU’s land and sea areas by 2030 and all degraded ecosystems by 2050 (European Commission 2025), whilst in the UK, the 25 Year Environment Plan includes goals to restore 75% of protected sites to favourable condition and the creation/restoration of 500,000 hectares of wildlife rich habitat outside of protected sites (Defra 2023).

## Supplementary Information

Supplementary file1 (DOCX 469 KB)

## Data Availability

The full collection of the georeferenced Second Land Utilisation maps will be available via the National Library of Scotland https://maps.nls.uk/series/second-land-utilisation-survey/. The 110 vectorised Second Land Utilisation maps used in this study will be free to download from the Environmental Information Data Centre (EIDC) https://eidc.ac.uk/—“Vector maps of the 1960 Second Land Utilisation Survey in England and Wales, from 110 published maps)”.
