# Supplementary material for: Assessing land use change and the impacts on semi-natural habitats across England and Wales using four time points between 1930 and 2020
Source: Landsc Ecol. 2025 Nov 20;40(12):220. doi: 10.1007/s10980-025-02189-8 (PMC12630304; doi:10.1007/s10980-025-02189-8)
Supplement: Supplementary file 1 — Supplementary file1 (DOCX 469 KB) [file 10980_2025_2189_MOESM1_ESM.docx]

Supplementary material

**Figure S1.** Availability of the 110 Second Land Utilisation Survey maps across England and Wales.


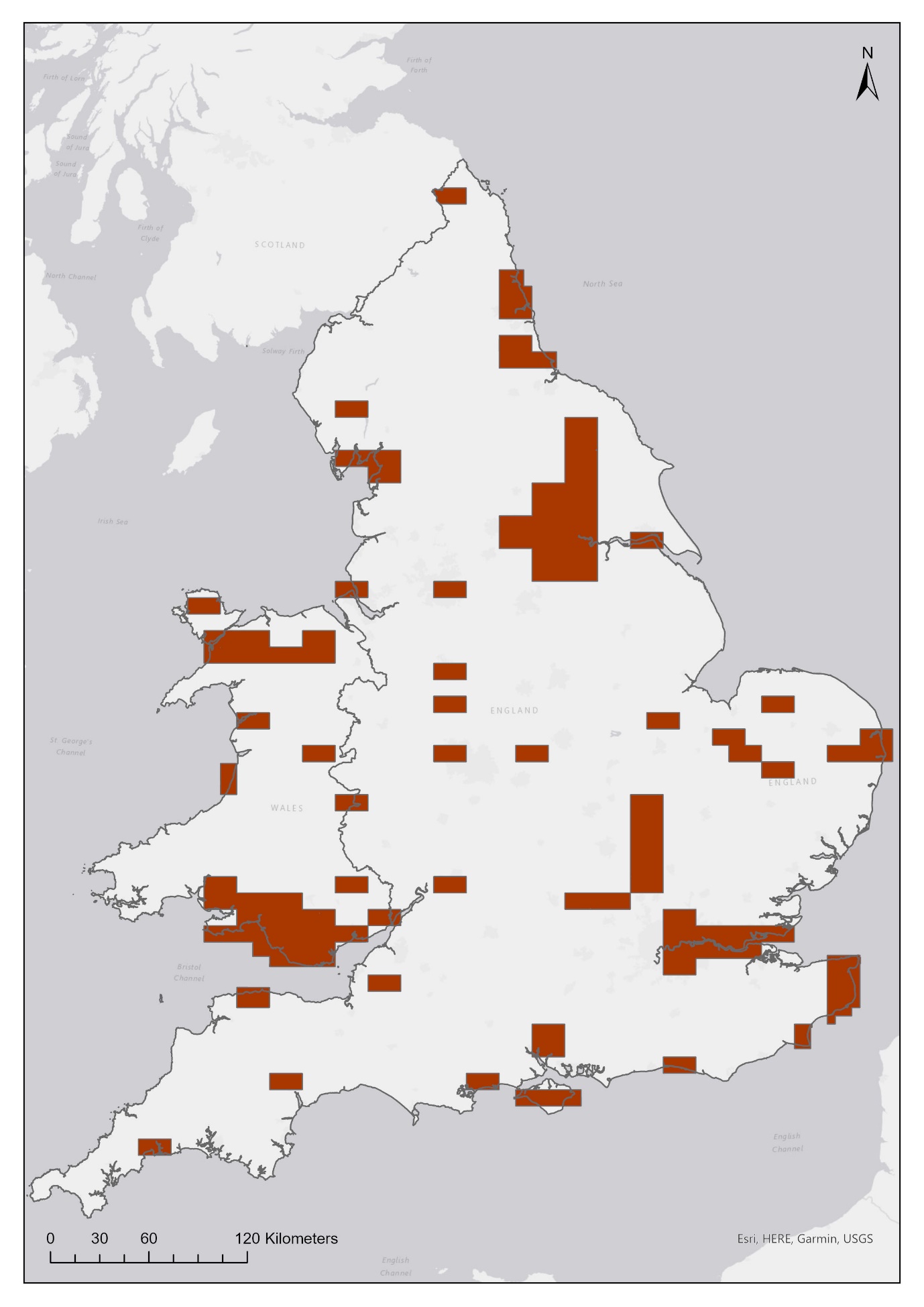


**Table S2.** Percentage cover of each broad category in 2020 found i) across the whole of England and Wales and (ii) the area covered by the 110 published Second Land Utilisation (2LUS) survey maps. The broad category was determined using the UKCEH Land Cover Map 2020 and was converted using Table 2.

| Broad category | England and Wales (%) | 2LUS maps (%) |
| --- | --- | --- |
| Broadleaved woodland | 8.5 | 9.3 |
| Coniferous woodland | 2.6 | 2.8 |
| Rough grassland, heath and wetland | 10.8 | 8.3 |
| Grassland | 33.8 | 28.8 |
| Arable and horticulture | 30.2 | 28.9 |
| Urban | 10.8 | 19.7 |
| Freshwater | 0.7 | 0.8 |
| Other | 2.5 | 1.5 |

**S3. Detailed methodology and validation for the digitisation of the Second Land Utilisation Survey**

*Pre-processing*

The first stage of the process involved scanning the 110 paper maps, which were each 89 x 58 cm in size. Maps were scanned at 300 dots per inch at a colour depth of 8 bits per channel (24-bit depth) and were saved as Tiff format images. This resolution is common for scanning large maps and is often a compromise between storage capacity, size of the map and resolution required for subsequent image analysis (Baily et al. 2011). Since the digital images contained no information on location, the second stage involved georeferencing them to the British National Grid coordinate system. This was achieved by relating coordinates of the 1 km grid background shown on the Second Land Utilisation Survey (2LUS) maps to the Ordnance Survey (OS) National Grid. This was performed using the ArcGIS Georeferencing tool with a second-order transformation. The average root mean square (RMS) error for this process was ca. 0.7 m. Finally, the georeferenced images were clipped to the map extent by removing the external frame and legend.

*Classification*

A semi-automated supervised classification method was developed to classify the different land use categories present in the 2LUS images. Coleman (1964) devised 13 main land use groups with distinct colour codes which are summarised in Table 1. Since the colour representing “market gardening” and “orchards” were the same, these land uses were combined for the classification. Firstly, the georeferenced 3-band images were converted to 16-bit single band images (256 colours). A manual look-up table was created for each map image identifying the dominant unique pixel values of the different land use classes. Because the maps were published by three different publishers, and slight colour variations were present, the same look-up table could not be applied to all map images. Any “noise” present on the images including text, contour lines and symbols were assigned a single blank value of 0. Similarly, pixel values which were common to multiple land use classes, such as the white colour were also assigned to 0. For these reasons it was not possible to classify both the “unvegetated land” and “derelict land” land use categories. The model reclassified the 256 pixels within a range of 0 to 10, representing the 10 land use categories and the noise category (pixel value = 0). The latter values were removed and replaced by applying a range of filtering and cluster models, all undertaken using ArcGIS v10.7.

*Post-processing*

The classified images were converted into a vector format prior to cleaning. Due to the issue of classifying white pixels, all “unvegetated land” was manually digitised, since these areas were incorrectly classified as both “arable” and “settlement”. Similarly, coastal areas which had complex markings over a white background, were often incorrectly classified as “arable”. To overcome this, a new land use category, “coastal” was formed. Extensive cleaning of the vectorised maps was undertaken, which took the form of basic GIS functions, such as eliminate using size-based rules, in addition to manual editing. This was essential for removing small areas of misidentified land classes, as well as blue and grey text, which has been classified as “water and marsh” and “settlements”, respectively. Railway lines, roads and very small rivers were inconsistently classified and were therefore removed, while “industry” and “transport” classes were grouped with “settlement” to ensure uniformity. “Derelict land” was often misclassified as “settlement”, so was later grouped with this category. Woodland was manually split into broadleaved and coniferous using the woodland symbology provided on the OS background of the 2LUS maps. The date of each OS basemap varied, however these were largely published or reprinted in the 1960s are thus consistent with the surveyed land cover data.

*Validation*

To validate the final vectorised 2LUS maps, 3,000 random points were generated across the 110 maps in England and Wales. The Mean High Water mark coastline boundary (Office for National Statistics 2021) was used to ensure points were not generated in the sea. For each point, a manual assessment was undertaken assigning the most representative land cover class to the point based on the raw georeferenced 3-band images. This work was undertaken by a researcher who was not involved in the classification and post-processing (ZO). The land cover class was also extracted from the classified 2LUS maps. From these two datasets, confusion matrices were generated for each of the eleven classes to evaluate the overall accuracy. For points which occurred on the boundary of two land use types, both types were captured and if either matched with the classified 2LUS maps they were assigned as correct.

The validation points had a good representation across the land use types (Table S3.1). The results are summarised in a confusion matrix (Table S3.2), where points along the main diagonal show complete agreement between the manually assigned points and the classified 2LUS maps. The classified 2LUS maps had an overall accuracy of 95.8%. Nine of the eleven classes had a producer’s accuracy greater than 90%. The most confusion was evident within the unvegetated land class, which required manual digitisation. Some of the confusion revealed for settlement class occurred due to points falling on roads, which were removed in the classified maps during the post-processing stage.

**Table S3.1** The proportion of each land use type found across the 110 published Second Land Utilisation Survey maps and the number of validation points occurring within each type.

| **Land Use Type** | **Number of validation points** | **Proportion (%)** |
| --- | --- | --- |
| Arable | 584 | 19.47 |
| Broadleaved and mixed woodland | 185 | 6.17 |
| Coastal | 63 | 2.10 |
| Coniferous woodland | 73 | 2.43 |
| Grassland | 1040 | 34.67 |
| Heath, moorland and rough land | 293 | 9.77 |
| Market gardening | 126 | 4.20 |
| Open spaces | 75 | 2.50 |
| Settlement | 463 | 15.43 |
| Unvegetated land | 31 | 1.03 |
| Water and Marsh | 67 | 2.23 |
| **TOTAL** | **3000** | **100%** |

**Table S3.2** Confusion matrix for the classified Second Land Utilisation Survey maps against 3,000 reference points.

|  | **Reference points (manual assignment)** | | | | | | | | | | | | |
| --- | --- | --- | --- | --- | --- | --- | --- | --- | --- | --- | --- | --- | --- |
| **Classified maps** | **Arable** | **Broadleaved woodland** | **Coastal** | **Coniferous woodland** | **Grassland** | **Heath, moorland & rough land** | **Market gardening & orchards** | **Open space** | **Settlement** | **Unvegetated land** | **Water and marsh** | **Total** | **User Accuracy** |
| **Arable** | 581 | 0 | 0 | 0 | 1 | 0 | 0 | 0 | 14 | 3 | 0 | 599 | **0.97** |
| **Broadleaved woodland** | 0 | 154 | 0 | 2 | 2 | 0 | 0 | 0 | 3 | 1 | 1 | 163 | **0.94** |
| **Coastal** | 0 | 0 | 63 | 0 | 0 | 0 | 0 | 0 | 0 | 2 | 2 | 67 | **0.94** |
| **Coniferous woodland** | 0 | 24 | 0 | 68 | 2 | 0 | 0 | 0 | 0 | 1 | 0 | 95 | **0.72** |
| **Grassland** | 1 | 5 | 0 | 1 | 1029 | 1 | 1 | 0 | 20 | 9 | 2 | 1069 | **0.96** |
| **Heath** | 1 | 0 | 0 | 2 | 2 | 292 | 0 | 0 | 3 | 0 | 0 | 300 | **0.97** |
| **Market gardening & orchards** | 0 | 0 | 0 | 0 | 2 | 0 | 124 | 0 | 2 | 1 | 0 | 129 | **0.96** |
| **Open space** | 0 | 0 | 0 | 0 | 0 | 0 | 0 | 74 | 1 | 1 | 0 | 76 | **0.97** |
| **Settlement** | 0 | 2 | 0 | 0 | 0 | 0 | 1 | 1 | 417 | 3 | 0 | 424 | **0.98** |
| **Unvegetated land** | 0 | 0 | 0 | 0 | 0 | 0 | 0 | 0 | 0 | 10 | 0 | 10 | **1.00** |
| **Water and marsh** | 1 | 0 | 0 | 0 | 2 | 0 | 0 | 0 | 3 | 0 | 62 | 68 | **0.91** |
| **Total** | 584 | 185 | 63 | 73 | 1040 | 293 | 126 | 75 | 463 | 31 | 67 | 3000 | **0.00** |
| **Producer Accuracy** | **0.99** | **0.83** | **1.00** | **0.93** | **0.99** | **1.00** | **0.98** | **0.99** | **0.90** | **0.32** | **0.93** | **0.00** | **0.96** |
| **Kappa** | 0.95 | | | | | | | | | | | | |

*References*

Baily, B., Riley, M., Aucott, P., & Southall, H. (2011). Extracting digital data from the first land utilisation survey of Great Britain - methods, issues and potential. Applied Geography, 31(3), 959–968. <https://doi.org/10.1016/j.apgeog.2010.12.007>

Coleman, A. (1964). Some Cartographic Aspects of the Second Series Land Use Maps. The Geographical Journal, 130(1), 167–170. <https://doi.org/10.2307/1794334>

Office for National Statistics (2023) Countries (December 2022) Boundaries GB BFC. In: <https://geoportal.statistics.gov.uk/datasets/ons::countries-december-2022-boundaries-gb-bfc/about>

**Figure S2.** Areas of the eight land cover classes within each of the five regions in England and Wales in the 1930s, 1960s, 1990 and 2020 (Total area assessed in the East = 2868.0 km^2^, Midlands = 1095.6 km^2^, North = 5797.5 km^2^, South = 4229.5 km^2^, Wales = 4610.2 km^2^). In 1930, broadleaved woodland and coniferous woodland are excluded, since these could not be distinguished.


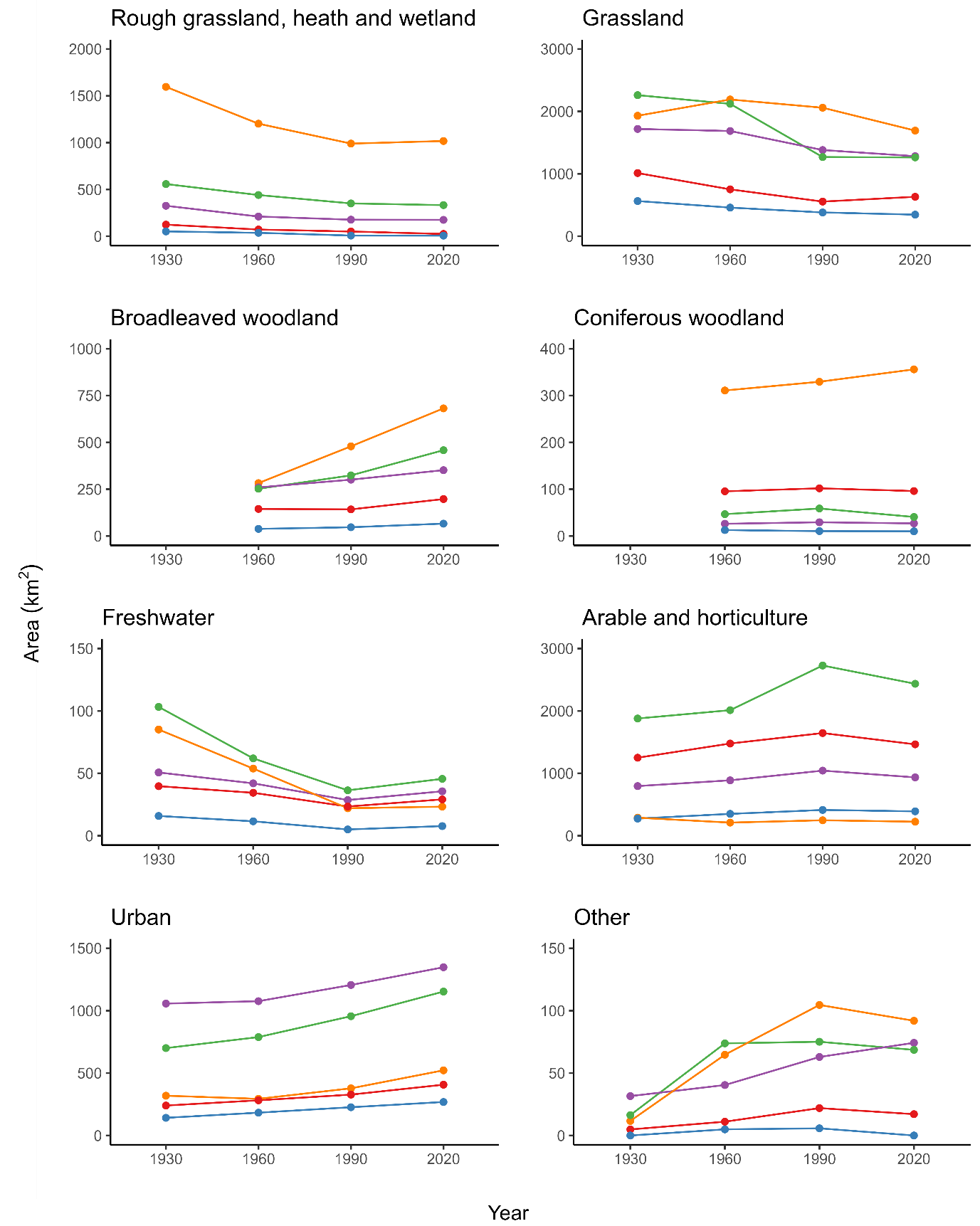


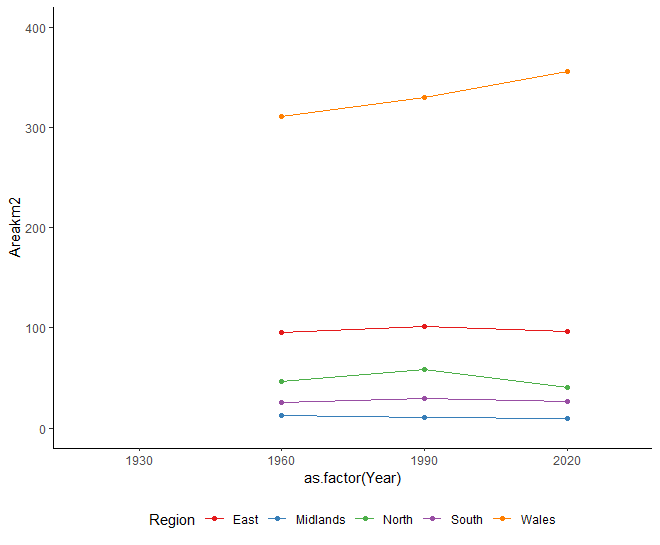


**Table S4** Known inaccuracies or uncertainties in the analysis of land use/land cover (LULC) change in 1930, 1960, 1990, 2020 across England and Wales.

| Issue | Description |
| --- | --- |
| Inconsistency between mapped airfields | Airfields were mapped as transport and hence urban land in the early time series maps, whilst airfields are sometimes classified as “arable” within LCM1990 and LCM2020, due to the spectral signature being very similar to bare ground. |
| Unvegetated land and coastal habitats not included in 1930 map | These broad categories were not included in the 1930 maps, and were grouped under an “other” category for the remaining time series. Any changes revealed in the “other” category should therefore be viewed with caution. |
| Inconsistency between small linear freshwater patches | Smaller linear patches of “freshwater” were mapped in 1930 and 1960. Whilst every effort was taken to remove these small patches that were often inconsistently mapped (e.g. middle part of river missing), some small linear rivers may remain, which would not have been mapped in the 1990 and 2020 datasets. |
| Classification of woodland type in 1960 | Broadleaved woodland and coniferous woodland were assigned using the OS backdrop after the image classification had taken place. The OS symbology was used to define these areas, and in some cases the line to delineate these two woodlands was unclear. It is therefore possible that some coniferous woodland was assigned to broadleaved woodland (including mixed woodland), if the coniferous tree symbol was in close proximity to a broadleaved tree symbol. |
| Manual correction of seawater | “Water and marsh” areas were manually split into seawater to correspond to the equivalent category in 1990 and 2020. |
